# Isolation and Identification of Endophytic Bacteria *Bacillus* sp. ME9 That Exhibits Biocontrol Activity against *Xanthomonas phaseoli* pv. *manihotis*

**DOI:** 10.3390/biology12091231

**Published:** 2023-09-12

**Authors:** Yating Feng, Yijie Zhang, Obaid Ullah Shah, Kai Luo, Yinhua Chen

**Affiliations:** 1 Sanya Nanfan Research Institute, Hainan University, Sanya 572025, Chinaobaidus890@gmail.com (O.U.S.);; 2 School of Tropical Agriculture and Forestry, Hainan University, Haikou 570228, China; 3 Collaborative Innovation Center of Nanfan and High-Efficiency Tropical Agriculture, School of Tropical Crops, Hainan University, Haikou 570228, China

**Keywords:** cassava, endophyte, *Bacillus subtilis*, bacterial blight, isolation, identification

## Abstract

**Simple Summary:**

Cassava is one of the most important crops worldwide. However, bacterial blight of cassava, as its most important disease, has caused substantial economic losses to the cassava industry. The main method of controlling cassava bacterial blight has been through chemical control, but this has polluted the environment significantly. In this context, biological control using microorganisms not only has the capacity to inhibit the disease, but also is environmentally friendly, showing great potential. Therefore, there is an urgent need to study the endophytic biological control method of cassava bacterial blight. On this basis, in the current study, we conducted a series of in vivo and in vitro experiments to isolate, screen and identify a biocontrol *Bacillus* ME9 with an antagonistic effect against *Xpm*11, the pathogen that causes cassava bacterial blight. In this study, the results also demonstrated that *B.* ME9 has a strong colonization ability, and revealed that its genome consists of a variety of genes related to antibacterial lipopeptides, which may be directly related to its antibacterial ability. *B*. ME9 had a strong antagonistic affect against *Xpm*11, and showed a certain effect on a variety of pathogens, which is expected to be further developed in its application as a commercial bacteriological agent.

**Abstract:**

In recent years, the bacterial blight of cassava has caused substantial economic losses to the Chinese cassava industry. Chemical control methods have become the primary approach to control this disease; however, their widespread usage and harmful residues have raised concerns about environmental pollution. In order to avoid this, it is urgent to seek a green ecological method to prevent and control it. Biological control through the utilization of microorganisms not only effectively inhibits the disease, but also gives consideration to environmental friendliness. Therefore, investigating an endophytic biological control method for cassava bacterial blight is of great importance. In this study, cassava leaf tissues were used as test specimens in order to isolate endophytic bacteria by using dilution and separation methods. *Bacillus* ME9, derived from cassava endophytic bacteria, exhibits good antagonism against a diverse range of pathogens, including *Xpm*11. Its genome consists of a series of genes encoding antibacterial lipopeptides, which may be directly related to its antibacterial capabilities. Furthermore, inoculation resulted in a substantial change in the diversity of the endophytic bacterial community, characterized by improved diversity, and displayed an obvious inhibition of pathogenic bacterial growth, demonstrating successful colonization within plants. The results laid a foundation and provided theoretical support for the development and utilization of cassava endophytic bacterial diversity and endogenous disease control strategies.

## 1. Introduction

Cassava (2n = 36, *Manihot esculenta* Crantz) originated in Brazil and is broadly planted in tropical regions. It is considered to be one of the most important global food crops [1], providing a staple supply of dietary calories, proteins, vitamins and micronutrients for over 700 million people, mainly in developing countries [2,3,4]. Cassava is an important starch plant that can be consumed directly as a staple food or processed into various starch products in regions lacking in caloric and other nutrition sources [5].

The production of cassava can be affected by many biotic factors. Cassava bacterial blight (CBB) is one of the most severe diseases worldwide, induced by the bacteria *Xanthomonas phaseoli* pv. *manihotis* (*Xpm*) [6,7]. Early symptoms are characterized by brown water-soaked angular spots appearing on the leaf tissue, occasionally surrounded by a chlorotic halo. These spots can affect the veins around them, making them discolored and frequently producing creamy white and later yellow-to-orange exudates on the lower side of the leaf. Blight results from spot coalescence, creating necrotic areas that become dry and curl the leaves. As the disease gradually worsens, *Xpm* invades the xylem vessels from the mesophyll and moves towards the stem through the petioles, which become brown and collapse. Vessel colonization in the stem allows *Xpm* to move systemically in plants. When infection reaches the upper and tender parts of the plant, stem rotting leads to dieback characterized by shoot apex wilting. New sprouts can grow from lower buds. However, if these buds are also contaminated by *Xpm* they will eventually wilt. Roots from highly susceptible cultivars can show the same symptoms, with discolored vascular strands surrounded by dry and rotten spots [8,9], which could lead to yield losses of 50% or more [10,11].

The most popular method to prevent plant diseases at present is chemical bactericides, which can be effective in the short term, but may damage the environment and even destroy the whole ecological environment over time [12]. In this context, beneficial endophytic bacteria were exploited to derive a potential antibiological inoculant to control diseases and alter chemical fungicides [13]. As reported previously, endophytic bacteria are bacteria that live inside plant tissues or cells, causing no visible damage to their host [14]. There are four ways an endophytic microbiome improves the growth and health of plants. Firstly, the endophytic microbiome produces antimicrobial metabolites and stimulates the defense responses of the plant. Secondly, the tolerance of the host towards abiotic stress may be enhanced by the endophytic microbiome. Furthermore, the endophytic microbiome helps plants improve their ability to absorb nutrients and releases growth-promoting phytohormones [15,16,17,18]. In a previous study, researchers found that through the modulation of local or systemic mechanisms and production of antioxidants counteracting plant ROS, abiotic stresses can be mitigated through the endophytic microbiome, a necessary internal partner of hosts. Zhou et al. (2021) [19] indicated that the seedling growth of *Pinus tabulaeformis*, under drought conditions, was improved after inoculating with the endophytic strain *Phoma* sp. Many endophytes have the capability to synthesize phytohormones such as auxins, cytokinins and gibberellins, affecting the plant hormonal balance [20,21]. Li et al. (2008) [22] isolated and screened endophytic bacteria from soybean root nodules and found that the screened strains essentially had the ability to produce IAA, and some of them could promote the dissolution of phosphate minerals and increase the utilization capacity of phosphorus by plants. Mageshwaran et al. (2022) [23] isolated three *B. subtilis* TRO4 strains from seven species of plants. CLO5 and PLO3 had strong antagonistic effects on *Solanomycetes*, *Rhodospora* and *Fusarium oxysporum*, and the antagonistic rates were all over 50%. At the same time, the production of the plant hormone IAA by TRO5 and CLO3 helped to promote plant growth. In recent decades, in order to be able to induce green and efficient plant productivity, the isolation of endophytic bacteria has been carried out in almost all known plants. It was proposed that the application of *B. amylolyticus* could reduce the incidence of Tomato spotted wilt virus (TSWR) in tomatoes by 80% and effectively inhibit the accumulation of Potato virus Y (PVY). Transcriptional analysis suggested that *B. amylolitica* could improve tomato disease resistance by inducing the SA-mediated defense gene [24]. The above results indicate that endophytic bacteria have potential functions in many fields, including the biological control of plant diseases.

Endophytic microorganisms of the *B.* genus are defined as strains with high biocontrol capacity. Several *B.* species have been identified as biological control agents and plant growth promoters. The biocontrol activities of *B.* against many common phytopathogens have also been detected in diverse studies [25,26]. In one previous study, *B.* cereus showed strong biocontrol potential against a great variety of plant pathogens, including the pathogenic bacteria of *Solanaceous* bacterial wilt, *Ralstonia solanacearum* [27]. Thanh et al. (2009) [28] showed that *B.* had good resistance to tomato bacterial wilt, potato fusarium wilt and black pepper foot rot, and under greenhouse conditions, the resistance to these three diseases was increased by 80% to 90% on average.

In addition to being able to enhance disease resistance, endophytic bacteria can also directly or indirectly affect the growth, biomass, quality and health of plants [29]. For instance, under salinity stress, the *Artemisia princeps Pamp* endophytic bacterial strain SAK1 was demonstrated to have the capacity to produce ACC deaminase and phytohormones that are able to promote the growth parameters of soybean plants [30]. Eke et al. (2019) [31] found that the endophytes *B. amyloliquefaciens* CBa_RR37 and *B. megaterium* CBm_RR10 in tomato showed strong drought tolerance, while tomato growth was increased based on their seed and root interaction. In addition, previous studies have proved that most *B.* have more than one growth-promoting function, such as the ability to produce phosphorus-soluble siderophores. The fresh weight and dry weight of *Mentha canadensis* also showed an increase after inoculation [32]. These results indicate that endophytic bacteria have the ability to improve plant disease resistance by promoting plant growth or enhancing their resistance to stress.

Compared to that for other important crops, the research about cassava endophytes is superficial. Furthermore, the potential of bacterial endophytes to control CBB is still unknown. Therefore, the objectives of this study were to isolate, screen and identify the cassava endophytes that have antagonistic activity against cassava wilt. At the same time, 16S rDNA technology was used to analyze the influence of cassava endophytes on bacterial wilt, which is expected to provide guidance for cassava production and planting in the field and provide a theoretical basis for subsequent research on cassava disease resistance mechanisms. This can both reduce the harm caused by bacterial wilt in cassava production and reduce the use of chemical pesticides.

## 2. Materials and Methods

### 2.1. Isolation and Screening of Antagonistic Bacteria

For isolation assays, tissue samples of cassava leaf were collected from the experimental base of the Haidian Campus of Hainan University, Hainan Province, China (20°06’ N, 110°33’ E). Methods for the surface sterilization of plant materials proposed by Coombs and Franco [33] were employed, with a few improvements for cassava endophytic bacteria isolation. Firstly, samples collected from the field were immediately saved in sterile packaging and sent to the laboratory. Then, the collected leaf samples were first washed with tap water, and then double-distilled sterile water was used to rinse the leaves well. The washed leaf samples were submerged in ethanol (75%) for 60 s and then in sodium hypochlorite (3%) for 60 s. After washing the treated plant samples with sterile water three times and cutting them into small fragments, they were pounded with a sterilized mortar and pestle. An aliquot of equal volume (100 μL) from each dilution (the tissue extracts obtained from leaf fragments were diluted by a factor of ten, from 10^−1^ to 10^−3^) was inoculated onto Luria Bertani (LB) agar, nutrient agar (NA) and tryptic Soyagar (TSA). The inoculated Petri plates were incubated for 48 h at 28 °C. Following, 100 μL of the last rinse in water, were plated on TSA medium and later observed for the appearance of colonies to test the sterilization results. The purified isolates were maintained on LB agar plates at 28 °C, and cells were stored in 50% (*v*: *v*) glycerol at −80 °C [34] for later studies.

### 2.2. Antagonistic Activity Assay against CBB

To evaluate and screen the endophytic bacterial isolates with antagonistic effects against cassava bacterial blight and verify a broad spectrum of bacteriostasis, we spread 100 μL isolated strain solution on LB plates for 2–3 d at 28 °C for subsequent preparation of agar discs. The *Xpm*11, *Escherichia coli* and *Staphylococcus aureus* isolates were cultured in LPGA and LB, respectively, until OD_600_ = 0.6, and *Bacillus amyloliquefaciens* (BA) was set as a control. Then, a 2 mL suspension of the pathogen strain was added to 198 mL of PSA medium (temperature was about 50 °C), mixed and poured into plates. Agar discs (8 mm in diameter) with a lawn of ME9 were cut, placed on test plates and incubated for 48 h at 28 °C. Discs without the bacterial isolate served as controls. Each control was set in triplicate, the zone of inhibition of each isolate was recorded in triplicate and the antagonistic ability for all bacterial isolates was evaluated [35].

Bacterial isolates were also tested on potato dextrose agar medium (PDA) for their antifungal activities against *Hevea brasiliensis* pathogens 1901 and *Stylopathic* bacteria W2, which had been isolated from preliminary laboratory tests. Of these, 1901 was isolated from diseased leaves of rubber, and W2 was derived from diseased plants of *Stylosanthes sinensis*. Four isolate agar discs were inoculated on the surface of the agar plate around the fungal disc, which was 2.5 cm away from the fungal disc. Antagonist activity was observed after incubation at 28 °C for up to 7 d. The value of inhibition was measured using the formula 100 × C T/C (T, treatment; C, control). Petri plates without bacterial isolates served as controls [36]. Each control was set in triplicate.

### 2.3. Morphology and Molecular Identification of Bacterial Isolates

Based on morphological characteristics [37], the bacterial strain with the strongest antagonistic activity against cassava bacterial blight was inoculated on an LB plate at 28 °C. Gram staining was carried out after inoculating for 18–24 h. Under the microscope, Gram staining and cell morphology were observed and photographed.

For molecular identification, first, the bacterial isolates’ genomic DNA was extracted using the Ezup Spin Column Bacterial Genomic DNA Mini-Prep Kit (Biomarker Biotech Beijing Co., Ltd., Beijing, China) for subsequent molecular identification. The PCR procedure and primers for 16S rDNA sequences were according to Fu and Wang [38,39]. The 16S rDNA amplicons were sequenced by Biomarker Biotech Beijing Co., Ltd., then compared using BLAST on the NCBI website, and the sequences with the highest homology to the target sequences were selected. The nucleotide sequences were analyzed using BLAST in GenBank to identify the strains.

OE Biotech Co., Ltd. (Shanghai, China) was chosen to conduct PacBio sequencing and analysis. Briefly, the Ezup Spin Column Bacterial Genomic DNA Mini-Prep Kit was used to extract ME9 genomic DNA. The genomic DNA was used for quality control through agarose gel electrophoresis and quantified using Qubit (Invitrogen, Waltham, MA, USA).

The genome was analyzed according to Flye and Canu [40,41]. Gene prediction of the assembled genome was performed using Prodigal (v2.6.3) [42]. tRNA and rRNA genes were predicted with tRNAscan-SE (v1.3.1) and RNAmmer (v1.2) [43], respectively. sRNAs were predicted using BLAST against the Rfam database [44]. Repeat sequences were predicted with RepeatMasker (v4.0.7) [45]. PILER-CR (v 1.06) [46] and CRT1.2-CLI [47] were used to predict the CRISPR sequence. Prophages were predicted with PhiSpy (v2.3) [48].

The putative genes were annotated for functional classification against databases according to Diamond [49], using the KEGG (Kyoto Encyclopedia of Genes and Genomes) and the GO (Gene Ontology) database, with an e-value of 1 × 10^−5^. A genome overview was created using Circos (v0.69) [50] to show annotation information.

### 2.4. Physiological and Biochemical Characterizations of Strain B. subtilis ME9

The endophyte ME9 was characterized though Gram stain, V-P, catalase, starch hydrolysis, methyl red, gelatin liquefaction, citrate and nitrate reduction tests according to the methods of Dong and Cao [51].

### 2.5. In Vivo Evaluation of the Effect of ME9 Isolates on the Development of CBB Disease

In this study, a plate confrontation experiment was performed to in vivo evaluate the antagonistic ability of ME9. The isolates were first added into LB medium and cultured at 200 rpm at 28 °C for 24 h for the preparation of bacterial suspensions, after which the culture broth was centrifuged at 12,000 rpm for 10 min, and the sediment was suspended in test tubes with 10 mM MgCl_2_. This process was repeated three times, and the final sediment was suspended in 10 mM MgCl_2_, followed by an adjustment to OD_600_ = 0.8. The pathogen *Xpm*11 was cultured in LPGA medium at 28 °C, and the OD_600_ was adjusted to 0.05 according to previous methods [52].

For spray assays, cassava plants at 30 d after planting (DAPs) were foliar-sprayed with 5 mL of the ME9 suspension. Two days later, *Xpm*11 and MgCl_2_ (negative control) were injected by syringe, and each split leaf had two injection ports on different sides, with *Xpm*11 on the right and MgCl_2_ on the left (control) [53]. The experimental design was completely randomized and two treatments were set, each consisting of 10 replications. After evaluating the disease incidence in hosts at 6, 9 and 12 d post inoculation with the pathogen, ImageJ was used to obtain the total area (TA) and affected area (AA) per leaf [54,55].

### 2.6. Effects of ME9 Inoculation on Endophytic Bacterial Community in Cassava Leaves

The surface washing of leaf samples was carried out with 70% ethanol solution three times, and they were then washed clean with distilled water. The samples were dried, 250–500 mg of fresh samples was transferred into 2 mL centrifuge tubes and the abrasive beads of the NucleoSpin tube were added to the sample tubes. The DNA was extracted using the MN NucleoSpin 96 s DNA kit (Tiangen Biotech (Beijing) T Co., Ltd., Beijing, China). The DNA concentration of the samples was measured using the Qubit dsDNA HS test kit and Qubit 4 fluorometer (Invitrogen, Thermo Fisher Scientific, Hillsboro, OR, USA) to ensure sufficient-quality microbiome DNA was obtained. Then, the V3–V4 region of the 16S rDNA gene in the obtained microbiome DNA was amplified using universal primers 335F (5′-CADACTCCTACGGGAGGC-3′) and 769R (5′-ATCCTGTTTGMTMCCCVCRC-3′) [56]. Both the forward 16S and reverse 16S primers ended with sample-specific sequences for deep sequencing. The reaction system was 10 μL, and the PCR amplification steps were as follows: DNA template 5–50 ng, *Vn F (10 μM) 0.3 μL, *Vn R (10 μM) 0.3 μL, KOD FX Neo Buffer 5 μL, dNTP (2 mM each) 2 μL, KOD FX Neo 0.2 μL, and ddH_2_O 10 μL; Vn F and Vn R were selected according to the amplification region. The next steps were denaturation at 95 °C for 5 min, denaturation at 95 °C for 30 s for 25 cycles, annealing at 50 °C for 30 s, extension at 72 °C for 40 s and finally at 72 °C for 7 min. All PCR amplicons were purified using Agencourt AMPure XP beads (Beckman Coulter, Indianapolis, IN, USA), Qubit dsDNA Customs assay equipment and the Qubit 4.0 fluorometer (Invitrogen, Thermo Fisher Scientific, Hillsboro, OR, USA) [57]. After the individual quantifying, amplicons were pooled in equal amounts. An Illumina Novaseq 6000 (Illumina, San Diego, CA, USA) was used for sequencing to construct the library.

### 2.7. Statistical Analysis

Analysis of bioinformation was performed through the BMK Cloud platform (Biomarker Technologies Co., Ltd., Beijing, China). Raw data were primarily filtered using Trimmomatic on the basis of the quality of single nucleotides [56] (v 0.33). Primer sequences were identified and removed using Cutadapt [57] (v 1.9.1). The PE reads obtained were assembled with USEARCH [58] (v 10), then chimeras were removed with UCHIME [59] (v 8.1). The high-quality reads generated from the above steps were the basis of the following analysis. The SILVA database [59] (release 132) was used to annotate OTU taxonomy based on the naive Bayes classifier in QIIME2 (v 1.8.0) [60] with a confidence threshold of 70%. QIIME2 and R software (v 4.0.2) were used to calculate and display the α-diversity, respectively. *β*-diversity, which was displayed using principal coordinate analysis (PCoA) and heatmaps, was calculated to evaluate the similarities of microbial communities from different samples using QIIME. Furthermore, linear discriminant analysis (LDA) effect size (LEfSe [58]) was also employed to test the significant taxonomic differences among groups, with the logarithmic LDA score set to 4.0 as the threshold for discriminative features. To explore the dissimilarities of the microbiome among different factors, a redundancy analysis (RDA) was performed in R using the package ‘vegan’.

## 3. Results

### 3.1. Isolation of Bacterial Endophytes from Cassava Leaves and Screening of Bacteria with Antagonistic Ability

**Figure 1:**
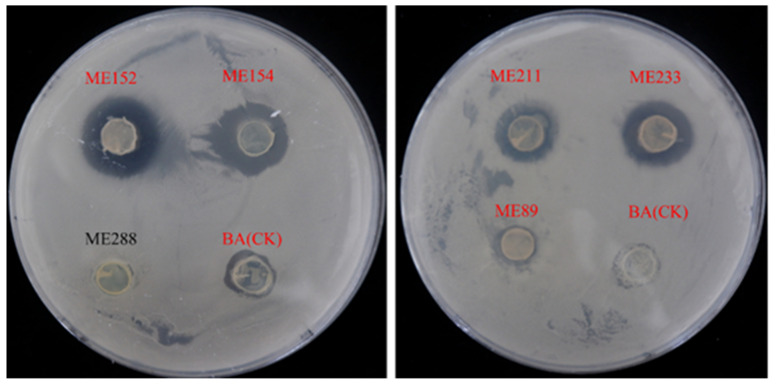
Antagonistic effect of some cassava endophytic bacteria on *Xpm*11 (red letters indicate that the strain had bacteriostatic ability).

A total of 304 strains of endophytic bacteria were isolated from cassava leaf tissue and these were named ME1-ME304. These isolates were chosen as test strains to perform in vitro antagonistic experiments against *Xpm*11, in which the different antagonistic abilities of these strains to *Xpm*11 were displayed on PSA plates. Among them, 28 endophytic bacteria were identified as strains antagonistic to *Xpm*11, which causes cassava bacterial blight (Table 1, Figure 1), and ME9 showed the strongest inhibiting ability against *Xpm*11, with the diameter of the zone reaching 12.06 ± 2.09 mm.

**Figure 2:**
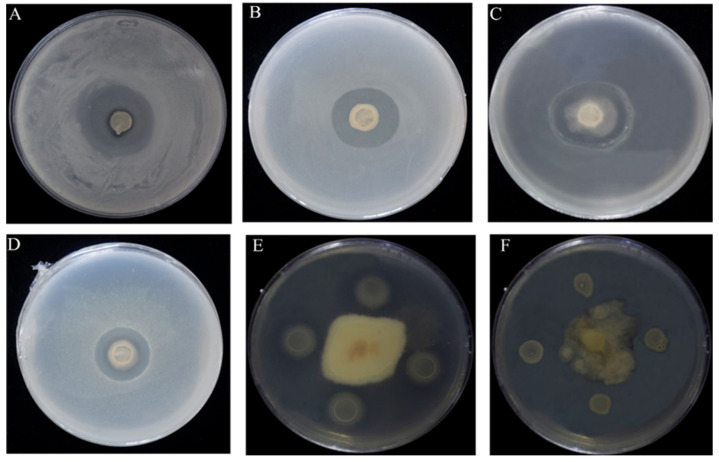
Strain ME9 antibacterial spectrum test results (**A**) *Xanthomonas phaseoli* pv. *Manihotis* 11; (**B**) *Xanthomonas phaseoli* pv. *Manihotis* 1; (**C**) *Escherichia coil*; (**D**) *Staphylococcus aureus*; (**E**) *Colletotrichum gloeosporioides*1901 (*Hevea brasiliensis*); (**F**) *Colletotrichum gloeosporioides* W2 (*Stylosanthes guianensias*).

The results also determined the inhibitory capacity of ME9 against a variety of other pathogens to determine its broad antibacterial spectrum. ME9 showed a great antagonistic effect on other pathogenic bacteria, such as *Xpm*1, *Staphylococcus aureus* and *Escherichia coli*, and on the fungi 1901 and W2 (Figure 2). Therefore, ME9 was chosen as an optimal antagonistic strain for further study.

### 3.2. Morphological and Molecular Identification of the Optimal Antagonistic Strain ME9

**Figure 3:**
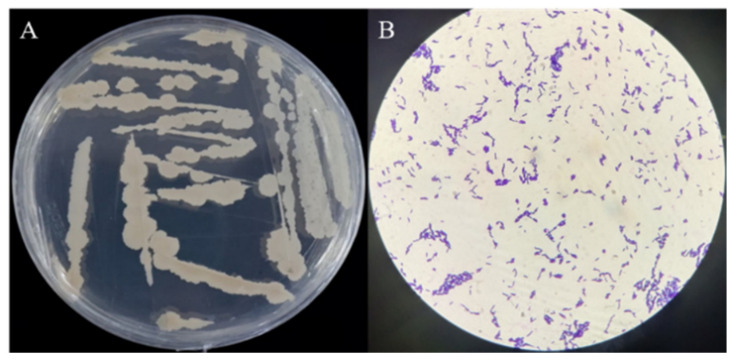
Colony morphology and Gram staining of strain ME9. (**A**) Colony morphology of strain ME9 on LB plate; (**B**): Gram staining of strain ME9 (100× magnification).

The isolated strain ME9 was characterized by studying physiological and biochemical characteristics that could indicate its capacity for plant colonization and potential as a biocontrol agent and plant growth promotor. The colony of strain ME9 on LB plates appeared white, round, larger, opaque, non-viscous and easy to pick out, the edge was not neatly round in the different periods of growth and the longer the culture time, the drier the colony surface became after 5 d of incubation at 28 °C (Figure 3A). Strain ME9 was a short, rod-shaped and Gram-positive bacterium under a microscope at 100× magnification (Figure 3B). The strain could carry out V-P and nitric acid reduction reactions and could liquefy gelatin. It had the ability to hydrolyze starch, it could make the methyl red indicator turn red, the results of citrate and indole tests were negative, the optimal pH was 8 and the salt tolerance was 9% (Table 2). According to Bergey’s manual of systematic bacteriology [61], the characteristics of the endophytic antagonist bacteria ME9 were similar to those of *B.*.

In addition, the analysis showed that the 16S rDNA sequence of ME9 was more than 99% identical to that of *B. subtilis* (CP070485.1) (Table 3). Therefore, based on the morphological characteristics and 16S rDNA analysis, ME9, which showed the strongest antagonistic effect, was identified as *B. subtilis*.

### 3.3. Analysis of ME9 de Novo Genome Sequencing

**Figure 4:**
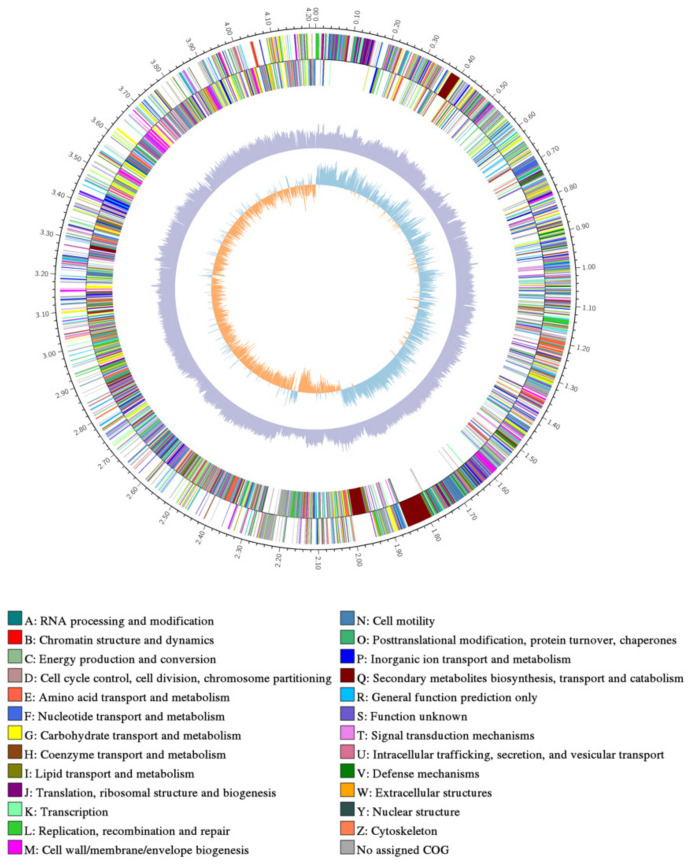
Genome map of strain ME9.From inside to outside are GC skew, GC content and non-coding RNA (rRNA is red, tRNA is blue, sRNA is green).

After sequencing and assembly, as shown in Figure 4, the genome-wide overview of strain *B. subtilis* ME9 showed a ring chromosome with a total assembly length of 4,215,379 bp and content of G + C 43.51%, consisting of one contig, 4,215,379 N_50_ and 4,215,379 L_50_. A total of 4225 protein-coding genes were identified, and 85 tRNA genes and 30 rRNA genes were predicted using tRNAscanSE (v1.3.1) and Barrnap (v0.8). The genomic characteristics of *B. subtilis* ME9 were compared with those of two previously reported *B. subtilis* strains, BYS2 and TY-1 (Table 4). The comparison indicated that the numbers of tRNA and rRNA in ME9 were basically the same, demonstrating that there was no difference in the protein synthesis ability of the different *B. subtilis* strains. However, compared with the other two strains, the number of protein-coding genes (CDS) of ME9 was greater than those of BYS2 and TY-1. That is, ME9 had more abundant functions and could have a greater impact on plant growth and development.

**Figure 5:**
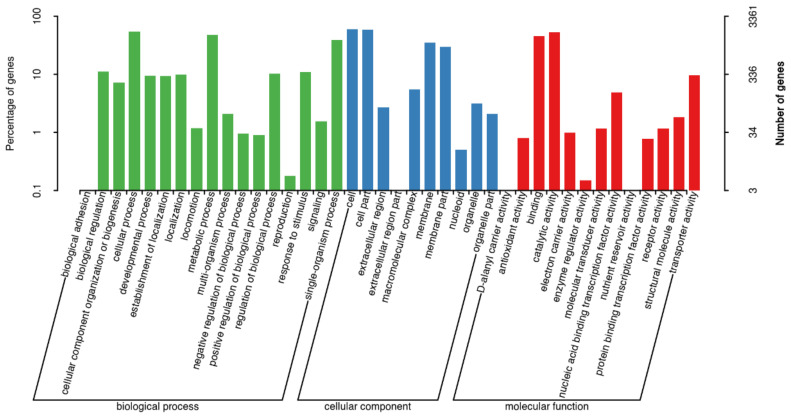
GO functional annotation on the genome of strain ME9 (red: molecular function; blue: cellular component; green: biological process).

Therefore, it was important to perform the genome annotation of strain ME9. In strain ME9, 3,361 genes were annotated in the GO database, and these were mainly divided into molecular functions (red), cellular components (blue) and biological processes (green). Parts of cell components totaled 10 types of functional genes, while cell and cell part genes accounted for more. There were 11 types of genes in the catalytic function classification, and the number of genes related to catalytic activity and binding was higher. Sixteen types of genes were annotated for the functions of biological processes, with a relatively high proportion of the genes involved in metabolic and cellular processes (Figure 5).

**Figure 6:**
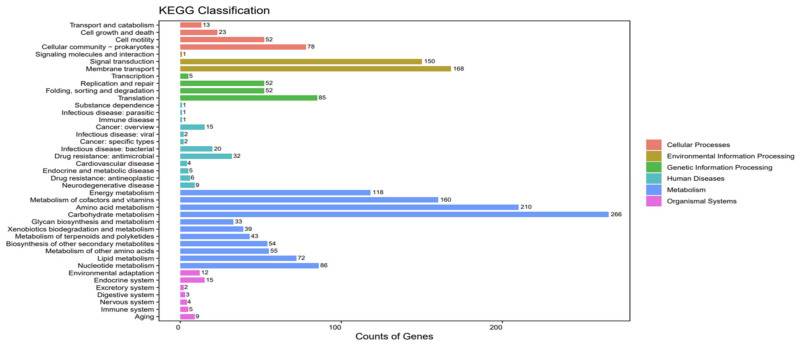
KEGG functional annotation of strain ME9 genome.

A total of 2462 ME9 genes were annotated in the KEGG database, as shown in Figure 6. The predicted metabolic pathways consisted of six categories: cellular processes, environmental information processing, genetic information processing, human diseases, metabolism and organismal systems, which contained 4, 3, 4, 12, 11 and 7 subclasses, respectively, mainly involved in carbohydrate metabolism (266 kos), amino acid metabolism (210 kos), signal transduction (168 kos), metabolism of cofactors and vitamins (160 kos) and other metabolic processes.

The antimicrobial active substances of *B.* are closely related to the secretion function of secondary metabolites. After the analysis of the ME9 genome using antiSMASH (v4.1.0), it was predicted that 30 gene clusters were related to the biosynthesis of secondary metabolites. Potential metabolites encoded by these gene clusters included citrulline biosynthetic (citrulline), sublancin 168 biosynthetic (antimicrobial peptide), subtilosin A biosynthetic, bacillomycin biosynthetic, bacillaene, fengycin, bacillibactin, surfactin, teichuronic acid biosynthetic, Sporulation_killing_factor_skfA_biosynthetic and zwittermycin A biosynthetic. At the level of 100% similarity, there were matching gene clusters in polyenes, fongenin, bacillus and Sporulation_killing_factor_skfA_biosynthetic synthesis. In addition, one gene cluster was associated with surfactant peptide synthesis, with a similarity of 82%, and two gene clusters were associated with citrulline and Zwittermycin A biosynthetic synthesis, albeit with a similarity of only 18%. Eighteen dissimilar biosynthetic gene clusters were compared in the antiSMASH database, which may be new secondary metabolite biosynthetic gene clusters (Table 5).

### 3.4. Endophytic Bacteria Strain ME9 Controlled Bacterial Wilt Disease

**Figure 7:**
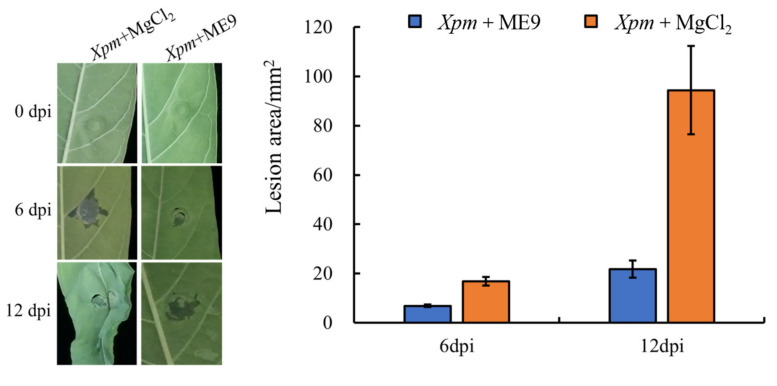
Effect of ME9 on cassava disease resistance. Note: **left**: effect of ME9 on phenotype of cassava bacterial blight after *Xpm* inoculation; **right**: comparative analysis of lesion area after spraying ME9.

A pot experiment was conducted in order to verify the antagonistic effect of the ME9 strain in living plants. After 48 h of culture, the ME9 bacterial solution with OD_600_ = 0.8 was induced to grow on cassava plants for about 30 d for an in vivo test to further verify the disease resistance of ME9 against CBB. The biocontrol strain ME9 was sprayed on, and the pathogen *Xpm*11 was inoculated via injection into the third and fourth leaves at the top of the cassava plant. After inoculation, the plants were cultured at room temperature with 12 h of light and 12 h of darkness. The results showed that ME9 could effectively inhibit the spread of disease spots (Figure 7). The leaves sprayed with ME9 bacteriological solution only showed small lesions at the inoculation point, and some split leaves showed a little chlorosis near the inoculation point after 12 d. The leaves treated with aseptic MgCl_2_ showed larger water-stained lesions, and the leaves near the inoculation site were obviously wilted. Thus, ME9 demonstrated a certain inhibitory effect on cassava bacterial blight.

### 3.5. Analysis of Changes in Structure of Cassava Endophytic Bacterial Community after Spraying ME9

A total of 1,433,377 pairs of reads were obtained from 18 leaf samples, including leaves sprayed with ME9 and leaves not sprayed with ME9, and 1,429,213 clean reads were generated after quality control and filtering of double-ended reads. At least 72,567 clean reads were obtained for each sample, with an average of 79,401. The rarefaction curve tended to flatten out (Figure S1), indicating that the sequencing depth was sufficient to illustrate the overall endophytic bacterial community structure of each cassava sample.

A total of 32 phyla, 85 classes, 221 orders, 442 families and 884 genera were detected in unsprayed samples, while 29 phyla, 77 classes, 202 orders, 394 families and 793 genera were detected in the samples with ME9 spraying (Table S1). After spraying ME9, the diversity of each classification level showed a slight decrease.

**Figure 8:**
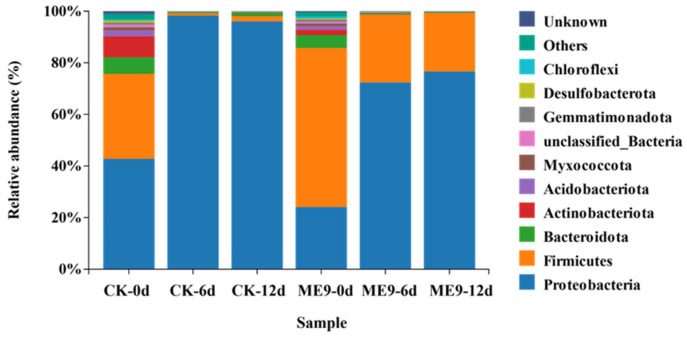
Principal coordinate analysis of endophytic bacteria under different treatments.

From the endophytic bacterial taxonomic analysis at the phylum level, the relative abundances of the 10 most abundant phyla (>1% of relative abundance in at least one sample) are shown in Figure 8. The endophytic bacterial flora of cassava leaves was mainly composed of Proteobacteria and Firmicutes. The abundance of Proteobacteria was 24.08% at 0 d, 72.35% at 6 d and 76.63% at 12 d. In the control group, the abundance of Proteobacteria was 42.74% at 0 d, 98.64% at 6 d and 96.06% at 12 d. The abundance of Proteobacteria showed an increasing trend as time went by in both the treatment and control samples. The abundance of Firmicutes was 66.16% at 0 d, 26.31% at 6 d and 22.69% at 12 d, and the abundance gradually decreased with the extension of the inoculation time. In the control group, the abundance of Firmicutes was 32.96% at 0 d, 0.96% at 6 d and 1.98% at 12 d. In the early stage of inoculation, the abundance showed a significant decrease. When comparing the same inoculation period, at 0 d of inoculation, the abundance of Proteobacteria was higher in the control group, while that of Firmicutes was greater with ME9 spraying. After 6 and 12 d of inoculation, the same trend was seen, with more Proteobacteria being found in the control group and the abundance of Firmicutes higher after spraying with ME9. These results indicated that ME9 application greatly affected the community structure of endophytic bacteria in the leaves and changed the abundance composition of each phylum.

**Figure 9:**
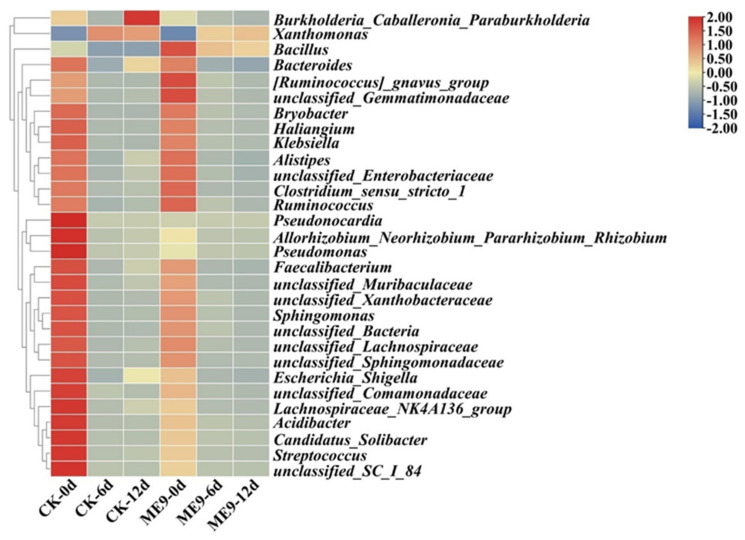
Bacterial community composition heatmap based on genus level between different treatments.

Analysis of the significant differences in bacterial community structures between the treatments was conducted on the basis of a relative abundance heatmap analysis of the 50 most abundant classified genera (Figure 9). After inoculation with ME9, the abundance of *Xanthomonas* in the treatment group significantly decreased with the extension of inoculation time, while the abundance of *B.* significantly increased. This indicated that the *B.* genus of ME9 had a certain colonization ability in leaves after spraying, while there was also an inhibitory effect on the *Xanthomonas* genus of *Xpm*11.

### 3.6. Analysis of Changes in Diversity of Cassava Endophytic Bacterial Community after Spraying ME9

**Figure 10:**
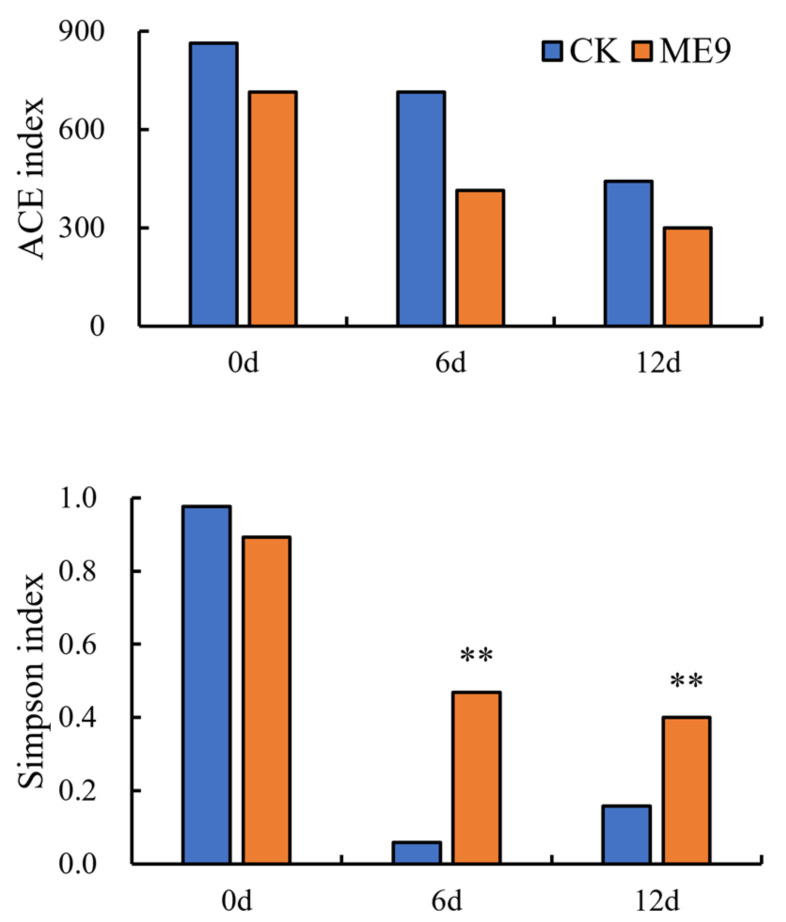
Analysis of bacterial α diversity among different treatments. (** <0.05).

The population of bacteria, the bacterial richness (ACE) and the bacterial Simpson index were used to estimate the abundance and α-diversity of endophytic microbiomes, and a statistical analysis was performed using either with or without ME9 spraying as explanatory variables. The *α*-diversity of endophytic bacteria in cassava leaves was analyzed (Figure 10), where the ACE represented the abundance of the endophytic bacteria community, while the Simpson index represented both the abundance and evenness of the endophytic bacteria colony. The results showed that after ME9 spraying, the ACE index of endophytic bacteria in cassava leaves decreased compared with the control group, and gradually decreased with the extension of time, indicating that ME9 spraying can reduce the abundance of endophytic bacteria in cassava leaves. The Simpson index of the treatment group was lower than that of the control group only in the early stage, and it was significantly higher than that of the control group at 6 and 12 d of spray treatment, indicating that ME9 treatment could make the community structure of cassava endophytic bacteria more uniform.

**Figure 11:**
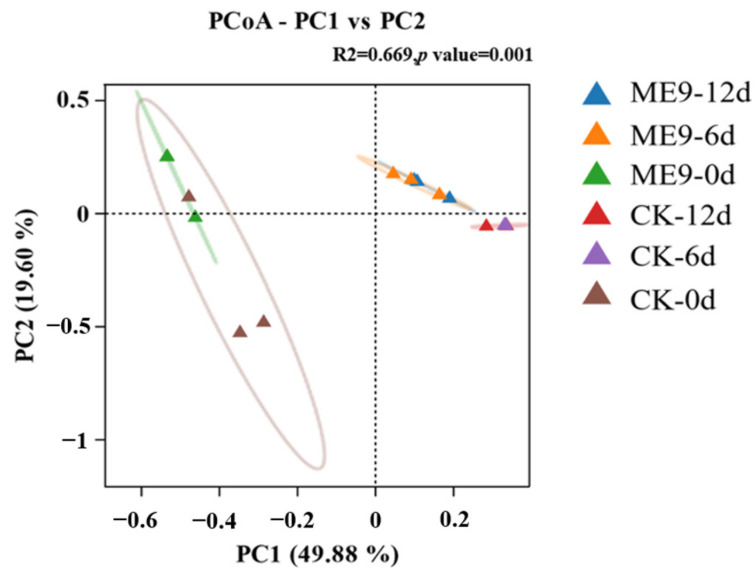
Composition of bacterial community based on phylum level between different treatments. Note: different colors represent the community structure distribution treated with ME9 (or not) after 0, 6 and 12d.

PCoA was used in this study to explain the changes in endophytic bacterial *β*-diversity in cassava leaves after ME9 spraying. As shown in Figure 11, PCoA results were obtained for cassava leaves after ME9 spraying and without ME9 spraying after different inoculation periods (0, 6 and 12 d). PCoA indicated that little obvious separation appeared between the samples in the treatment and control groups at the early spraying stage (0 d), showing that there was no significant difference between the samples. However, at the late spraying stages (6 and 12 d), there was a significant difference in the distribution of the samples, and the samples after ME9 treatment were concentrated in the first quadrant. The samples of the untreated control group were distributed in the fourth quadrant (*p* < 0.05), indicating that ME9 application significantly affected the *β*-diversity of endophytic bacteria in cassava leaves at the later stages.

### 3.7. Bacterial Groups with Significant Differences after Spraying ME9

**Figure 12:**
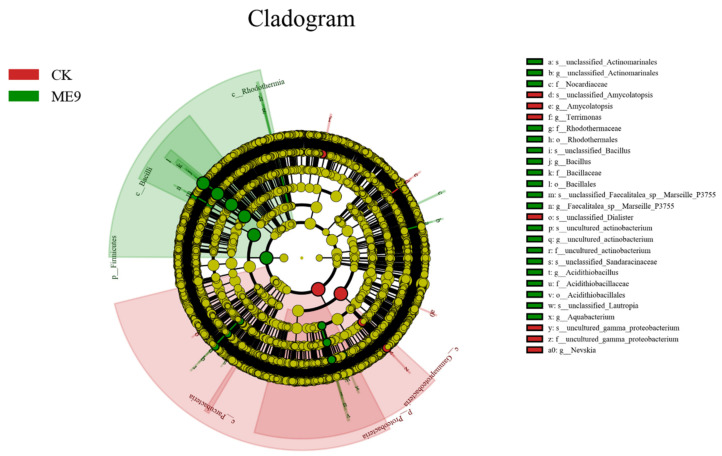
LEfSe of bacteria with significant differences among different treatments (yellow: the species with no significant differences; green: characteristic strains of the experimental group (ME9); red: characteristic strains of control group (CK)).

**Figure 13:**
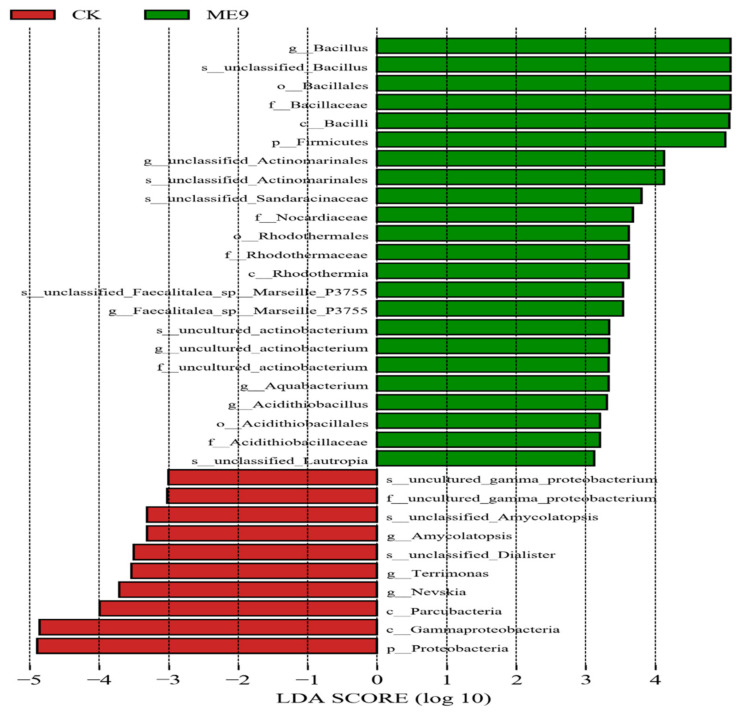
LDA analysis of endophytic bacteria in different treatments.

After comparing the microbial communities to identify the specialized bacterial groups after spraying with ME9 or not and characterizing the microbial diversities, the bacterial community data were analyzed using LEfSe at different levels. These results can establish the statistical significance, biological consistency and effect-size estimation of predicted biomarkers [64]. In the experiment, the default logarithm LDA value 4.0 was taken as a fixed value. When the value was greater than 4, it indicated that the strain had significant differences and could be used as a marker strain (Figure 12). A total of 33 distinct bacterial groups were identified (Figure 13). At the phylum level, Firmicutes and Proteobacteria were labeled when ME9 was applied and not applied, respectively. At the genus level, the microflora after ME9 spraying was characterized by the presence of *B.*; there were no labeled strains in the samples that were not sprayed with ME9. These results indicated that ME9 can colonize cassava leaves.

**Figure 14:**
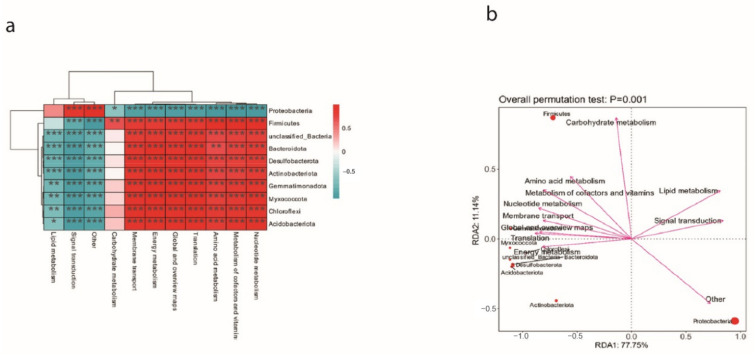
(**a**) Correlation analysis of cassava dominant bacteria and metabolic pathways. (**b**) RDA redundancy analysis of cassava dominant flora and metabolic pathways. (* <0.05, ** <0.01 and *** <0.001).

The influence of metabolites on microbial communities was checked after spraying ME9. In order to study the potential metabolic function of the dominant flora inoculated with ME9, a correlation analysis was conducted between its metabolic pathways and the dominant phyla. It was found that among the top 10 most abundant phyla, Proteobacteria-related pathways showed a significantly negative correlation compared with other pathways, and only the lipid metabolism and signal conversion pathways were positively correlated with them (Figure 14a). Through RDA redundancy analysis, it was found that Firmicutes showed a significant positive correlation with other phyla in the glucose metabolism pathway. The Proteobacteria showed a significant negative correlation (Figure 14b).

## 4. Discussion

Cassava, one of the three key tuber crops in the world, is an especially important crop in tropical regions [1]. Millions of people, mainly in developing tropical countries, use it for a staple supply of essential nutrients for the human body, such as starch, vitamins and calories [2,3,4]. The production of cassava is affected badly by cassava bacterial blight (CBB), which is caused by the bacteria *Xanthomonas phaseoli* pv. *manihotis* (*Xpm*) [6,7]. This disease is mainly prevented by chemical pesticides, which can damage the environment and affect ecology [13]. Biocontrol agents are a kind of control agents that are friendly to the environment and can control disease effectively [14]. In this study, a strain of *B.* that can effectively control cassava bacterial wilt was isolated from cassava by means of a plate test, and its genome information was analyzed, while its disease resistance was verified through in vivo and in vitro tests.

### 4.1. Endophytic Microorganisms, an Essential Part of the Plant Microbiome

Many endophytic isolates with antagonistic activity are common in plants, such as *Pseudomonas*, *Pantoea* and *B.* [65,66,67,68,69]. They secrete a variety of metabolites and thereby enhance resistance to a variety of pathogens. Among them, *B.* is the most widely distributed and it can be isolated in rhizosphere soil and plant tissues [70]. *B.* sp. have usually been defined as endophytic microbiota in many plants, where they protect plants from disease and promote their growth under certain conditions [71].

In previous studies, many *B.* sp. have been isolated from cassava, an economic food crop in the tropics, including from the roots, leaves and stems [5,72,73]. Some isolates from cassava show abilities to improve the utilization rate of phosphorus in the rhizosphere, produce indole acetic acid, siderophores, phytases, organic acids, ACC deaminase, cyanogens, lytic enzymes and oxalate oxidases, and solubilize various sources of organic and inorganic potassium and zinc [5,72]. In addition, they have shown antagonistic effects in vitro against *Macrophomina phaseolina, Fusarium oxysporum, F. solani, Sclerotinia sclerotiorum, Rhizoctonia solani* and *Colletotricum* sp. [72]. Many similarly interesting features were observed in the *B. subtilis* strain ME9, including biocontrol ability in the form of antagonism against *Xpm*11 (Figure 2).

### 4.2. Metabolic Characteristics of Endophytic Microorganisms Are Key in the Development of Cassava

The rhizosphere is the main place where microorganisms exist; endophytic microorganisms, which usually exist in plant tissues, are also widely present in farm soil [74]. Therefore, host plants are to some extent affected by the soil environment during their period of development and growth. In this process, many rhizosphere carbon sources are available to metabolize, making microorganisms compete for the metabolites compounded by plants [66].

Most of the carbohydrates, almost 40–60%, that plants fix through leaves are secreted through plant roots [75]. These exudates consist of low-molecular-weight compounds (amino acids, organic acids and sugars, among others) and high-molecular-weight compounds (mucilage and proteins), which are crucial to rhizosphere microbial communities in soil. This study determined the metabolic capacity of ME9, which was mainly able to metabolize carbohydrate and amino acid sources (Figure 6). Through correlation analysis with metabolic pathways (Figure 14), the abundance of carbohydrate-metabolism-related pathways in the cassava endophytic bacteria community after ME9 application was shown to be higher, which may be due to the physiological and biochemical translation of the plant itself under the influence of the bacteria.

In addition to providing the plant with a defense response, carbon can indirectly affect the plant’s immunity through the hormone signaling network within the plant’s immune system. It can also promote the rapid lignification of the cell wall in the early stages of plant disease, inducing the expression of certain PR proteins or increasing the amounts of flavonoids secreted and increasing plant disease resistance [76]. Pathogens may also affect the proportion of soluble sugar and single-component sugar in plants through pathogen transformation and utilization, involving the formation of cell structure and inhibiting plant photosynthesis. The influx of large amounts of sugar into the reservoir from the transformation of the infection site compensates for the sugar loss, leading to changes in sugar levels in the infected tissue under the influence of different plant pathogen interactions [77]. Through effector mediation and pathogen molecular pattern triggering, sugar signaling molecules can directly participate in the immune response and regulate the immune function of plants. Studies have shown that there are a wide variety of sugar signaling molecules in plant defense responses, including trehalose, sucrose, pinobiose, galactosol, etc., which can improve the ability of plants to resist pathogens by influencing the expression of defense-related genes, promoting the synthesis of secondary metabolites such as flavonoids, and activating the activity of various protein kinases (such as mitogenic-activated protein kinase MAPK). However, the signaling mechanism is still unclear [78,79,80].

Plant defense responses to pathogens can also be regulated by the interaction of sugars with plant hormones [81], such as cell wall invertases (CWIs), which is induced by ABA [82]. In addition to its role as a sensor in glucose research experiments, hexokinase (HXK1) can also participate in the glycolysis pathway as an enzyme to promote the conversion and utilization of glucose [83]. In addition, in hypersensitivity (HR), the expression of resistance-related genes (PR) is also achieved by programmed cell death mediated by hexokinase [84]. For example, *OsSWEET11* in rice can be directly regulated by the TAL effector *PthXO1*, which is secreted by rice bacterial fusarium wilt itself [85]. All these indicate that ME9 spraying could further enhance the immune capacity of cassava by regulating sugar metabolism.

Other important characteristics of the *B. subtilis* strain that help improve plant growth and provide protection were detected, including catalase, nitrate reductase, citrate, amylolysis and indole acetic acid production (Table 2). Nitrate reductase is essential for plant growth. It was found that nitrate can change the composition of cell biofilm, and the increase in nitrate reductase concentration can improve the membrane permeability and its composition and increase the content of extracellular polysaccharides and proteins on the membrane. The above results indicate that improving the assimilation and utilization efficiency of nitrate can promote the growth of plants and indirectly enhance plant disease resistance [86,87]. In addition, the defensive systems of plants could be enhanced by the bioagents, by increasing the antioxidant genes, which would eventually increase plant growth and yield production [88].

Therefore, the metabolic characteristics of the *B. subtilis* ME9 strain isolated in this study endow it with the ability, to some extent, to stimulate plant growth. It can directly or indirectly affect plants’ growth, and thereby improve their disease resistance.

### 4.3. The biocontrol Capacity of B. Subtilis Could Be Associated with Its Ability to Produce Lipopeptides

Among all the genes of the *B. subtilis* genome, about 4–5% of them were related to antibiotic synthesis, indicating that this strain shows great potential for the production of more than 20 varieties of antimicrobial compounds [89]. The lipopeptide system is considered the most important biocontrol capacity in many species of the *B.* genus [90,91]. Lipopeptides exist in bacteria and have great importance not only in colonization processes but also in the induction of host resistance responses. Lipopeptides consist of surfactin, iturin and fengycin [89]. The lipopeptide measurement of ME9 showed the same capacity to produce these compounds (Table 5).

Biosurfactants are important lipid peptides related to the activity of *B. subtilis*. Pathogenicity can be inhibited by integrating surfactants into the lipid bilayers of cell membranes and impeding their integrity [89]. The activity of plant pathogens may be affected by bacillibactin, showing growth inhibition; for example, bacillibactin can produce inhibitory effects against *P. syringae* in vivo or in vitro. Bacillibactin also upregulates specific genes that influence microbial interactions [92]. Bacillomycin biosynthetic is a polyene antibiotic that can inhibit prokaryotic protein synthesis [93]. In addition to working directly, lipopeptides can improve the ability to resist pathogens by triggering plant defenses. Farace et al. (2015) [94] found that surfactins and mycosubtilin (a member of the iturin family) were able to improve the resistance of grapevine seedlings to infection caused by *B. cinerea*, and had the capacity to activate defense genes in the plant, minimizing the damage caused by this fungus [94]. The role of these substances in ME9 is important for understanding how the disease resistance mechanism of ME9 works, and it still needs to be studied further.

## 5. Conclusions

In recent years, research on the development of green and sustainable agricultural systems has begun to focus on biological control, and more and more biocontrol strains have been developed and utilized [25,26,27]. Among these microorganisms, endophytic bacteria have been favored by researchers because of their strong stability, diversity and control effect. In this work, the *B. subtilis* strain ME9 was isolated from cassava leaves. This strain has great potential for inhibiting the growth of many pathogens, especially *Xpm*11, suggesting it for use as a kind of sustainable biocontrol agent. Through genome sequencing, a large number of synthetic gene clusters for antibacterial substances were found in the genome of strain ME9, indicating that strain ME9 has the potential to synthesize antibiotics.

In addition, after ME9 spraying, the bacterial community structure in cassava leaves was changed, and the abundance of *Xanthomonas* decreased significantly with the extension of inoculation time, while the abundance of *B.* increased significantly. These results indicated that the ME9 strain belonging to the *B.* genus had certain colonization abilities in leaves after spraying, while also having an inhibitory effect on *Xpm*11.

As cassava is considered an important food crop in tropical areas, this could be the beginning of the discovery of endophytic microorganisms in the plant itself, offering great biocontrol potential for the tropical agricultural industry. In the future, the disease resistance mechanism of the *B.* sp. ME9 in cassava requires further research, and optimized screening conditions should be employed to screen for a target strain with growth-promoting and disease resistance effects even more conductive to high yields and the sustainable production of cassava.

## Figures and Tables

**Table 1:** Antagonistic effect of 28 endophytic bacteria against *Xpm*11.

Strain Number	Bacteriostatic Zone Width (mm)	Antagonistic Effect
BA (CK)	3.86 ± 0.62	+
ME2	9.40 ± 1.59	++
ME9	12.06 ± 2.09	+++
ME13	5.16 ± 1.35	++
ME21	5.49 ± 0.51	++
ME3	3.60 ± 0.84	+
ME5	5.17 ± 1.74	++
ME52	3.88 ± 0.32	+
ME59	0.58 ± 0.18	+
ME62	2.25 ± 0.73	+
ME81	1.48 ± 0.90	+
ME85	2.26 ± 0.74	+
ME97	1.24 ± 0.21	+
ME126	2.38 ± 0.77	+
ME139	2.33 ± 1.08	+
ME142	4.64 ± 0.86	+
ME148	1.55 ± 0.35	+
ME152	5.2 ± 0.38	++
ME154	5.13 ± 0.77	++
ME159	4.52 ± 1.05	+
ME160	3.03 ± 1.02	+
ME221	6.34 ± 0.31	++
ME222	2.98 ± 0.47	+
ME233	3.80 ± 0.27	+
ME234	1.55 ± 0.67	+
ME262	3.82 ± 0.31	+
ME267	1.83 ± 1.13	+
ME269	2.50 ± 0.76	+
ME278	3.56 ± 0.52	+

Note: “+++” means 10 mm < bacteriostatic zone width < 15 mm; “++” means 5 mm < bacteriostatic zone width < 10 mm; “+” means bacteriostatic zone width <5 mm.

**Table 2:** Some physiological and biochemical characteristics of strain ME9.

Test Item	Result
Gram stain	+
Catalase	+
Methyl red	+
V-P	+
Gelatin liquefaction	+
Nitrate reductase	+
Citrate	–
Amylolysis	+
Indole reaction	–
Salt tolerance	
3%	+
5%	+
7%	+
9%	+
12%	–
pH	
3	+
4	+
5	+
6	+
7	+
8	+
9	+
10	–
11	–

Note: + positive, – negative.

**Table 3:** Molecular identification of endophytic antagonistic bacteria.

Strain	Closest Type Strain	Reference Sequence	Taxonomy (Genus)	Identity (%)
ME2	*Bacillus velezensis*	CP090905.1	*Bacillus*	100.00
ME9	*Bacillus subtilis*	CP070485.1	*Bacillus*	100.00
ME13	*Bacillus cereus*	AP023005.1	*Bacillus*	100.00
ME21	*Brevibacillus laterosporus*	CP032410.1	*Brevibacillus*	99.93
ME3	*Bacillus altitudinis*	MN543858.1	*Bacillus*	100.00
ME5	*Bacillus licheniformis*	MT368012.1	*Bacillus*	100.00
ME52	*Curtobacterium oceanosedimentum*	NR_104839.1	*Curtobacterium*	98.38
ME59	*Leclercia adecarboxylata*	KJ184954.1	*Leclercia*	95.18
ME62	*Bacillus subtilis*	KM083800.1	*Bacillus*	99.13
ME81	*Pantoea agglomerans*	KX233853.1	*Pantoea*	87.54
ME85	*Bacillus* sp.	JF783985.1	*Bacillus*	97.31
ME97	*Pantoea agglomerans*	KX233853.1	*Pantoea*	87.54
ME126	*Bacillus subtilis*	MK824707.1	*Bacillus*	99.34
ME139	*Leclercia adecarboxylata*	MF716711.1	*Leclercia*	99.02
ME142	*Leclercia adecarboxylata*	MN314177.1	*Leclercia*	81.48
ME148	*Lelliottia amnigena*	MH127568.1	*Lelliottia*	97.32
ME152	*Curtobacterium albidum*	MN889264.1	*Curtobacterium*	99.35
ME154	*Bacillus subtilis*	CP070485.1	*Bacillus*	100.00
ME159	*Bacillus subtilis*	KY621524.1	*Bacillus*	98.13
ME160	*Bacillus subtilis*	OK314473.1	*Bacillus*	96.92
ME221	*Bacillus subtilis*	KF641841.1	*Bacillus*	97.14
ME222	*Pseudomonas fulva*	CP014025.1	*Pseudomonas*	100.00
ME233	*Lelliottia amnigena*	MH127568.1	*Lelliottia*	97.32
ME234	*Pseudomonas oryzihabitans*	MN889339.1	*Pseudomonas*	98.96
ME262	*Bacillus subtilis*	AB210968.1	*Bacillus*	99.05
ME267	*Pantoea rodasii*	MN036531.1	*Pantoea*	98.97
ME269	*Bacillus subtilis*	MN704432.1	*Bacillus*	98.22
ME278	*Bacillus subtilis*	OM320445.1	*Bacillus*	97.82

**Table 4:** The genomic characteristics of strain ME9 were compared with model strains.

Genomic Features	ME9	*B. subtilis* BYS2	*B. subtilis* TY-1
Genome size (bp)	4,215,379	4,030,791	4,030,269
Total number of contigs	1	1	1
G + C content (%)	43.51	43.88	43.88
Protein coding genes	4225	3914	3960
rRNAs	30	30	30
tRNAs	85	86	86
Reference	This study	[62]	[63]

**Table 5:** AntiSMASH annotation functional classification of *B. subtilis* ME9.

Cluster ID	Type	Start	End	Similar Cluster	Similarity (%)	Gene No.
Cluster1	Sactipeptide-Head_to_tail	203,539	226,492	Sporulation_killing_factor_skfA_biosynthetic	100	23
Cluster2	Nrps	356,566	421,957	Surfactin biosynthetic	82	42
Cluster3	Cf_putative	430,220	445,726	-	-	15
Cluster4	Cf_putative	792,277	811,041	-	-	13
Cluster5	Cf_putative	905,411	926,022	-	-	21
Cluster6	Cf_fatty_acid	1,082,363	1,103,340	-	-	18
Cluster7	Cf_putative	1,105,596	1,118,226	-	-	13
Cluster8	Cf_putative	1,122,458	1,130,297	Zwittermycin A biosynthetic	18	7
Cluster9	Terpene	1,149,536	1,170,339	-	-	21
Cluster10	Cf_fatty_acid	1,197,815	1,218,753	Citrulline biosynthetic	18	20
Cluster11	Cf_putative	1,285,185	1,309,397	-	-	23
Cluster12	Nrps-Transatpks-Otherks	1,768,293	1,878,119	Bacillaene biosynthetic	100	51
Cluster13	Nrps	1,934,123	2,017,555	Fengycin biosynthetic	100	41
Cluster14	Terpene	2,091,767	2,113,665	-	-	19
Cluster15	Glycocin	2,259,120	2,279,290	Sublancin 168 biosynthetic	100	25
Cluster16	T3pks	2,296,555	2,337,652	-	-	43
Cluster17	Cf_putative	2,464,156	2,473,581	-	-	10
Cluster18	Cf_putative	2,780,797	2,789,215	-	-	9
Cluster19	Cf_putative	3,087,983	3,097,411	-	-	9
Cluster20	Cf_saccharide	3,147,556	3,177,167	-	-	20
Cluster21	Nrps	3,260,110	3,309,851	Bacillibactin biosynthetic	100	43
Cluster22	Cf_putative	3,443,950	3,450,117	-	-	7
Cluster23	Cf_saccharide	3,508,590	3,534,024	-	-	25
Cluster24	Other	3,583,412	3,624,158	-	-	37
Cluster25	Cf_saccharide	3,639,465	3,690,036	Teichuronic acid biosynthetic	100	40
Cluster26	Cf_putative	3,725,216	3,738,816	-	-	12
Cluster27	Sactipeptide-Head_to_tail	3,825,676	3,847,287	Subtilosin A biosynthetic	100	19
Cluster28	Sactipeptide-Head_to_tail	3,850,033	3,918,547	Subtilosin A biosynthetic	100	68
Cluster29	Cf_putative	3,998,714	4,004,500	Bacillomycin biosynthetic	40	6
Cluster30	Cf_putative	4,084,162	4,103,024	-	-	18

## Data Availability

The data generated and analyzed in this study are available in the Supplementary Materials.

## Supplementary Materials

The following supporting information can be downloaded at: https://www.mdpi.com/article/10.3390/biology12091231/s1, Figure S1: Dilution curves of sample; Table S1: Endophytic bacterial composition in different treatments.
