# Supplementary material for: Isolation and Identification of Endophytic Bacteria Bacillus sp. ME9 That Exhibits Biocontrol Activity against Xanthomonas phaseoli pv. manihotis
Source: Biology (Basel). 2023 Sep 12;12(9):1231. doi: 10.3390/biology12091231 (PMC10525512; doi:10.3390/biology12091231)
Supplement: Supplementary file 1 [file biology-12-01231-s001.zip › biology-2574923-supplementary.pdf]

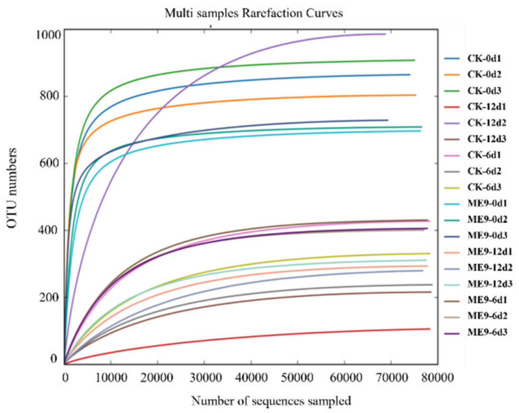

Figure S1 Dilution curves of sample

Table S1 Endophytic bacterial composition in different treatments

Treatment	Sample	Phylum	Class	Order	Family	Genus
Spraying no ME9	CK-0d	28	68	183	343	646
	CK-6d	25	92	142	235	397
	CK-12d	27	56	137	263	467
Spraying ME9	ME9-0d	27	66	171	317	582
	ME9-6d	23	58	139	259	456
	ME9-12d	24	47	122	222	398
